# Commissural Prolapse: Combining the Best From Two Aortic Valve Repair Techniques

**DOI:** 10.1093/icvts/ivag013

**Published:** 2026-01-08

**Authors:** Anze Djordjevic, Peter Juric, Suzana Danojevic, Miha Antonic

**Affiliations:** Department of Cardiac Surgery, University Medical Centre Maribor, 2000 Maribor, Slovenia; Department of Cardiac Surgery, University Medical Centre Maribor, 2000 Maribor, Slovenia; Department of Cardiac Surgery, University Medical Centre Maribor, 2000 Maribor, Slovenia; Department of Cardiac Surgery, University Medical Centre Maribor, 2000 Maribor, Slovenia

**Keywords:** aortic regurgitation, aortic valve repair, commissural avulsion, valve-sparing surgery, aortic root aneurysm, cardiopulmonary bypass

## Abstract

Aortic valve commissural avulsion is an exceptionally rare and often underdiagnosed cause of aortic regurgitation. We report a 30-year-old male with severe chronic aortic regurgitation and a mildly dilated aortic root, in whom intraoperative inspection revealed spontaneous avulsion of the right–noncoronary commissure. The patient underwent valve-sparing aortic root replacement using a combined approach: remodelling root replacement with external annuloplasty according to the Lansac technique, and targeted commissural reinforcement based on David principles. Postoperative and 6-month imaging showed stable root geometry, good leaflet coaptation, mild central regurgitation, and improved left ventricular function. This case highlights the diagnostic value of multimodality imaging and illustrates that tailored application of established valve-sparing techniques can achieve durable repair in rare commissural lesions.

## INTRODUCTION

Commissural avulsion of the aortic valve is a rare cause of aortic regurgitation (AR) and is most commonly associated with trauma, infective endocarditis, or aortic dissection.^1^ True “spontaneous” commissural detachment is seldom reported and often misdiagnosed preoperatively due to its overlap with more common aortic pathology.^2^ Transoesophageal echocardiography (TOE) and contrast-enhanced computed tomography (CT) imaging can assist in excluding dissection and suggesting structural valve abnormalities,^2^ yet definitive diagnosis is typically made intraoperatively. We present a young patient with chronic AR caused by spontaneous right–noncoronary commissural avulsion, successfully treated with a hybrid valve-sparing root reconstruction.

## CASE PRESENTATION

A 30-year-old otherwise healthy male presented with chest discomfort and progressive dyspnoea. Examination showed no signs of trauma, infection, or connective-tissue disease; there were no phenotypic or familial features suggestive of heritable aortopathy.

**Figure 1.**
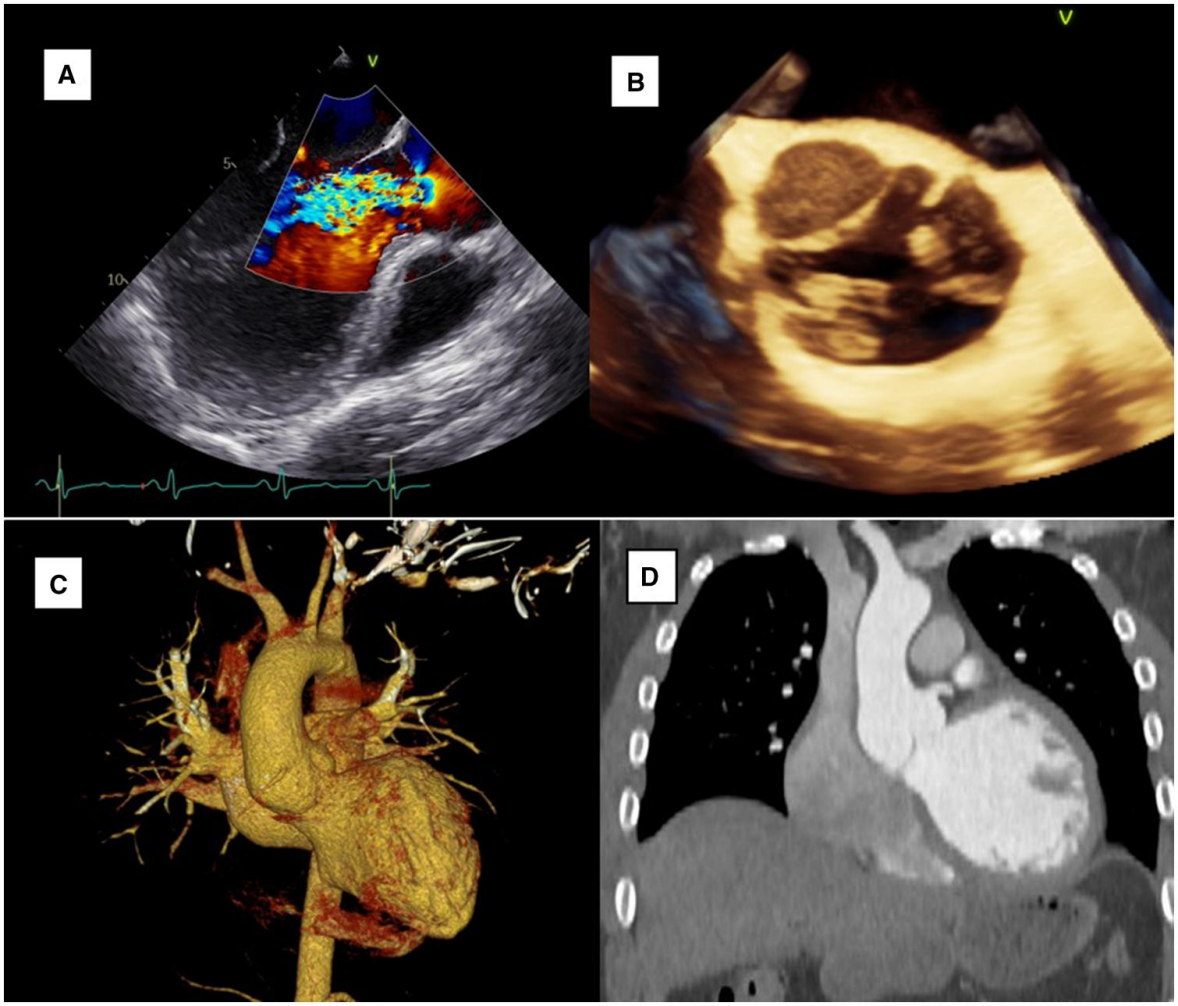
Preoperative Transoesophageal Echocardiography (TOE) and CT Imaging. (A, B) Mid-oesophageal long- and short-axis TOE showing severe eccentric aortic regurgitation and right–noncoronary commissural avulsion. (C, D) CT angiography confirming root dilation and excluding dissection.

Preoperative TOE revealed severe eccentric AR due to loss of coaptation between the right and noncoronary cusps, with LV dilation (end-diastolic diameter 66 mm) and reduced ejection fraction (35%) (**Figure 1A and B**). CT angiography excluded dissection and showed a mildly dilated aortic root (40 mm) (**Figure 1C and D**). Medical stabilization included beta-blockers and angiotensin-converting enzyme (ACE) inhibition, appropriate for chronic AR without acute haemodynamic compromise.

**Figure 2.**
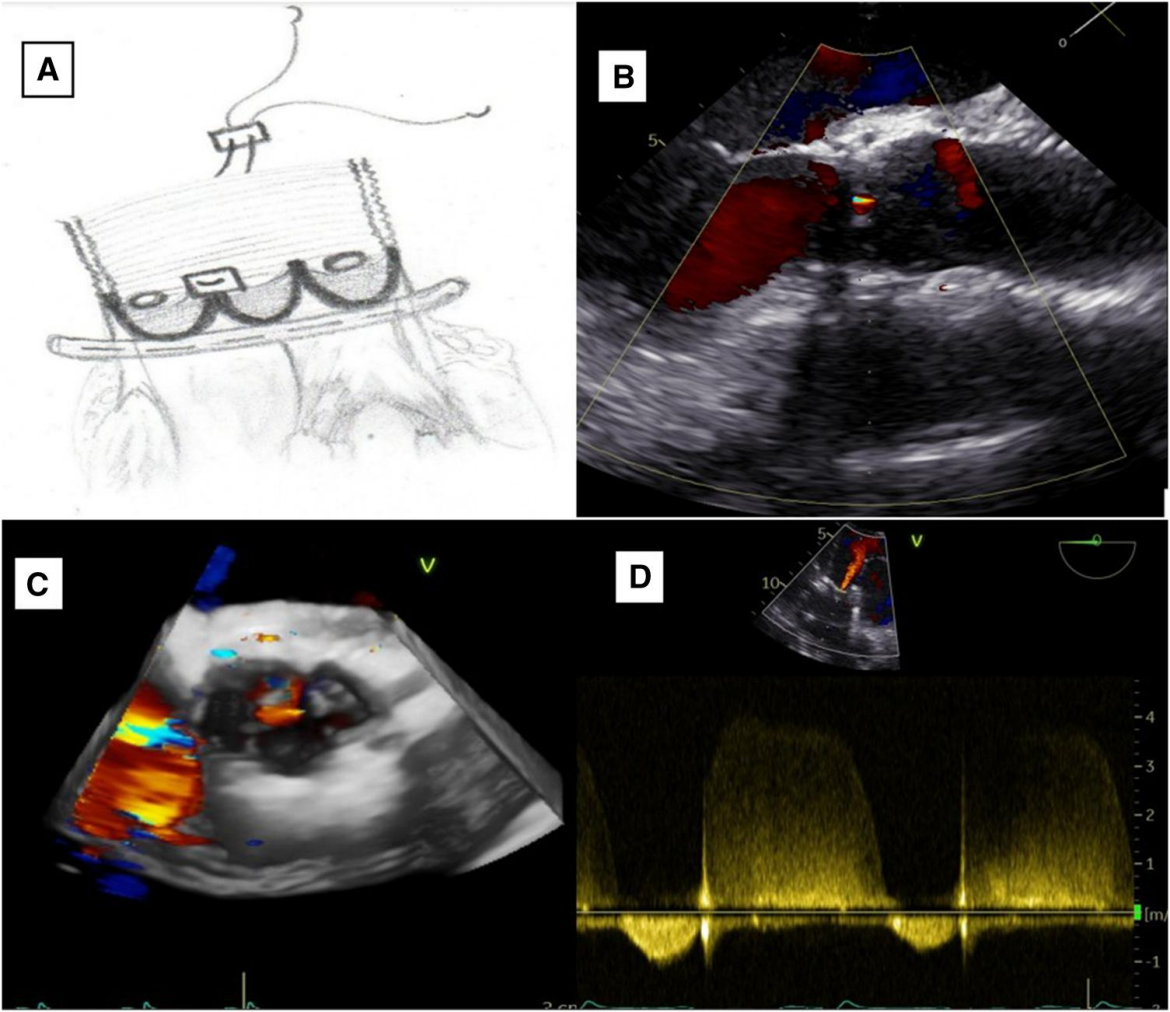
Schematic and Postoperative Transoesophageal Echocardiography (TOE). (A) Completed remodelling root reconstruction with external annuloplasty and commissural reinforcement. (B–D) Postoperative TOE demonstrating restored coaptation with mild central aortic regurgitation.

Surgery was performed through median sternotomy under cardiopulmonary bypass. After resection of the sinuses of Valsalva, the annulus measured 25 mm and a 28 mm Cardioroot graft (Intervascular SAS, La Ciotat, France) was selected. A 29 mm Coroneo external annuloplasty ring (CORONEO Inc., Montreal, Canada) was implanted with 6 pledgeted subvalvular sutures following Lansac principles.^3^ Intraoperative inspection identified avulsion of the right–noncoronary commissure. Commissural reattachment was accomplished with 4–0 polypropylene pledgeted mattress sutures, restoring commissural height, and symmetry^4^ (**Figure 2A**). Coronary reimplantation and distal graft anastomosis completed the root reconstruction. Intraoperative TOE demonstrated trivial residual AR and symmetrical cusp motion. The excised aortic wall appeared macroscopically normal; histopathology and genetic testing were not performed.

Recovery was uneventful. Echocardiography at discharge showed mild central AR, ejection fraction (EF) improved to 40%, and left ventricular end-diastolic diameter (LVEDD) decreased to 63 mm (**Figures 2B-D**). At 6-month follow-up, the patient was asymptomatic (New York Heart Association (NYHA) functional class I) with preserved valve competence, EF 45%-50%, no recurrent prolapse, and stable root geometry. He is enrolled in structured long-term surveillance with annual clinical and echocardiographic assessments.

## DISCUSSION

Commissural avulsion is a rare and underrecognized mechanism of AR, with most reported cases occurring in association with trauma, endocarditis, or type A dissection.^1^^,^^2^^,^^5^ Few cases of spontaneous or “secondary” commissural detachment have been described, and diagnosis is often delayed because preoperative imaging may not clearly distinguish avulsion from cusp prolapse or root dissection. In this patient, multimodality imaging helped exclude life-threatening pathology and suggested structural valve injury, although definitive diagnosis was possible only during operative inspection.

Valve replacement has traditionally been favoured in commissural avulsion due to concerns regarding tissue quality and uncertainty regarding durability of repair. However, in young patients with preserved leaflet integrity and localized pathology, valve-sparing strategies can provide excellent functional outcomes and avoid long-term prosthesis-related complications. In this case, the cusps were pliable and structurally intact apart from the avulsed commissure, supporting a repair-first approach.

Our operative strategy combined 2 established principles: remodelling of the aortic root with external annuloplasty^3^ to restore annular and sinotubular geometry, and direct commissural reinforcement using pledgeted sutures based on David reimplantation principles.^4^ This hybrid technique was chosen not as a novel operation but as a pragmatic adaptation tailored to the specific pathology. External annuloplasty provided annular stabilization that may protect against late dilation, while commissural reattachment corrected the primary regurgitant mechanism. The case illustrates that optimal valve-sparing surgery requires flexibility in combining techniques rather than strict adherence to a single established approach.

Although the root measured only 40 mm, localized thinning and asymmetry identified intraoperatively justified root replacement despite being below classical replacement thresholds. Current guidelines allow individualized decision-making when tissue quality is abnormal or valve-sparing repair would otherwise be at risk.

Six-month follow-up demonstrated preserved valve competence, improved ventricular function, and stable root geometry. Nonetheless, long-term outcomes of commissural avulsion repair remain unknown due to the rarity of this pathology. Continued clinical and echocardiographic surveillance is essential to monitor durability and better define prognosis in similar cases.

## CONCLUSION

Spontaneous or secondary commissural avulsion should be considered in unexplained AR, particularly in young patients. Multimodality imaging aids diagnosis, but intraoperative inspection remains definitive. A tailored hybrid valve-sparing approach can restore competence with excellent early outcomes. Long-term surveillance is essential to assess durability.

## Data Availability

The data underlying this article are available in the article and in its online supplementary material. All included figures have been created by the authors.
